# Comparison of Monolateral External Fixation and Internal Fixation for Skeletal Stabilisation in the Management of Small Tibial Bone Defects following Successful Treatment of Chronic Osteomyelitis

**DOI:** 10.1155/2017/6250635

**Published:** 2017-11-26

**Authors:** Yicun Wang, Hui Jiang, Zhantao Deng, Jiewen Jin, Jia Meng, Jun Wang, Jianning Zhao, Guojing Sun, Hongbo Qian

**Affiliations:** Department of Orthopedics, Jinling Hospital, School of Medicine, Nanjing University, Nanjing, China

## Abstract

**Background:**

To compare the salvage rate and complication between internal fixation and external fixation in patients with small bone defects caused by chronic infectious osteomyelitis debridement.

**Methods:**

125 patients with chronic infectious osteomyelitis of tibia fracture who underwent multiple irrigation, debridement procedure, and local/systemic antibiotics were enrolled. Bone defects, which were less than 4 cm, were treated with bone grafting using either internal fixation or monolateral external fixation. 12-month follow-up was conducted with an interval of 3 months to evaluate union of bone defect.

**Results:**

Patients who underwent monolateral external fixation had higher body mass index and fasting blood glucose, longer time since injury, and larger bone defect compared with internal fixation. No significant difference was observed in incidence of complications (23.5% versus 19.3%), surgery time (156 ± 23 minutes versus 162 ± 21 minutes), and time to union (11.1 ± 3.0 months versus 10.9 ± 3.1 months) between external fixation and internal fixation. Internal fixation had no significant influence on the occurrence of postoperation complications after multivariate adjustment when compared with external fixation. Furthermore, patients who underwent internal fixation experienced higher level of daily living scales and lower level of anxiety.

**Conclusions:**

It was relatively safe to use internal fixation for stabilization in osteomyelitis patients whose bone defects were less than 4 cm and infection was well controlled.

## 1. Introduction

Chronic infectious osteomyelitis is one of the most challenging conditions an orthopaedic surgeon should be faced [1] and is closely associated with bone loss and soft tissue defect [2]. In order to remove all necrotic and infected bone, aggressive skeletal debridement is usually conducted, which often results in significant segmental defects and requires complicated reconstruction [3]. Hence, bone reconstruction and bone fragment stabilization are needed [4]. Bone grafting is the most common technique for the reconstruction of bone defect [5]. Specialized reconstructive procedures, such as Ilizarov technique [6] and induced membrane technique [7] are widely used in recent years. Furthermore, adjuvant therapy, such as bony defect fillers and antibiotic vectors, would be used during surgery [8, 9].

Surgeons tended to use reconstruction through bony arthrodesis as a reconstructive alternative to amputation, which consists of external fixation and internal fixation. Surgeons prefer to use external fixation over internal fixation when performing fusions [10, 11]. The controversy about internal fixation lies in that implanted hardware may incur recurrent infection and untimely hardware failure due to poor bone condition in the position of injury because of prior infection and multiple debridement procedures [12, 13]. However, external fixation can lead to a time-dependent incidence of complications, such as pin-tract infection, joint stiffness, and soft tissue irritation [14]. Moreover, external fixators are cumbersome for patients [15] and it needs consistent maintenance, which can be problematic in a population of noncompliant patients [16].

When selecting suitable procedures for bone grafting with chronic infectious osteomyelitis patients, surgeons should take into account the strength and weakness of each method. There have been series of studies comparing the advantage between internal fixation and external fixation in noninfected tibia nonunion patients [17–19]. However, in the case of chronic infectious osteomyelitis, few studies were conducted.

Hence, this study aimed to compare the salvage rate and related complications between internal fixation and external fixation with bone grafting in patients who had been controlled from active infection by treating with intravenous antibiotics and multiple debridement.

## 2. Material and Methods

### 2.1. Subjects

In this study, 125 patients with tibia bone defect caused by osteomyelitis were collected and retrospectively reviewed between January 2008 and December 2015. The diagnosis of osteomyelitis and infected nonunion was based on the standard published by the American Academy of Orthopaedic Surgeons [20], which included clinical symptoms, laboratory tests, imaging manifestations, and culture of specimens. Patients who had bone nonunion caused by chronic osteomyelitis for at least 3 months were enrolled in the present study. The exclusion criteria were as follows: patients suffering from pathologic fracture initially; patients whose bone defect was larger than 4 cm after debridement, which was not suitable for bone grafting according to the consensus published by Chinese Association of Orthopaedic Surgeons (CAOS) in 2016 [21]; patients whose surface skin was not integrity; patients who had persistent infection after debridement and antibiotic therapy; patients who had poor compliance. The selection of patients was briefly introduced in a flow chart (Figure S1). Informed consent was obtained from each patient and the privacy rights of patients were always observed. The study protocol of our research conformed to the ethical guidelines of the 1975 Declaration of Helsinki as reflected in approval by the local ethical committee.

### 2.2. Operation Procedure

All patients with chorionic osteomyelitis were underwent a staged operation procedure.

In the first stage, debridement was conducted. Radical debridement was conducted in all nonviable and infected tissues and the debridement proceeded until bleeding and viable bone (characterized by “paprika sign”) were present at the resection margins. Specimens of bone and purulent fluid were sent for aerobic and anaerobic cultures. Also, in order to rule out malignant changes, bone specimens were also sent for pathologic examination. In the end, the wound was irrigated with a copious amount of saline.

In the second stage, skeletal stabilization and antibiotic therapy were conducted. After radical debridement, bone defect was presented and skeletal stabilization was needed. An external fixator was applied to stabilize the bone defect, which was also necessary for controlling infection. Antibiotic therapy was conducted to control infection. Initially, antibiotic therapy which covered the most common pathogens was used. After the culture and sensitivity results were available, antibiotic regimen which specifically manages the infecting organism was applied instead. Intravenous and oral antibiotics were administered for 4 to 6 weeks. The status of infection was checked by clinical symptoms (swelling, hot, pain, redness, and so on), blood tests (C-reactive protein (CRP), erythrocyte sedimentation rate (ESR), white blood cells (WBC), and procalcitonin (PCT)), bone biopsy, and imaging manifestations (X-rays). No further construction was conducted when an active infection was presented.

After well controlling of infection, bone grafting was used to construct bone defect. The autogenous cancellous bone was harvested from iliac crest (bilateral or monolateral) and placed at defect site. Afterwards, either internal fixation or external fixation were used for skeletal stabilization. The selection of external or internal fixation for patients was based on patients' needs, wishes, and financial condition. The whole surgery procedure followed the consensus published by the American Academy of Orthopaedic Surgeons [20]. The limb was elevated after surgery and sutures were removed 2-3 weeks later. The short-term nonweight bearing exercise began on the second postoperative day on bed. The toe-touch weight bearing with the aid of two crutches began when osteotylus was shown in imaging manifestation and lasted 4–6 weeks, followed by increments to gradually achieve full weight bearing within 3 months.

### 2.3. Collection of Clinical Data

Baseline data consists of demographics, initial fracture pattern, method of initial fixation, time to graft after the first procedure, surgery time, donor site, length of bone defect, body mass index (BMI), blood albumin, fasting blood glucose (FBG), total cholesterol, triglyceride, and low density lipoprotein (LDL). Imaging data included plain radiographs and a CT scan of the affected limb. Each patient was evaluated prospectively by two surgeons.

Bone consolidation was followed up at 1.5, 3, 6, 9, and 12 months after operation and estimated using X-ray analysis. The rate of salvage, time to final union, and the occurrence of complications were collected. Clinical union was identified as full weight bearing without pain and radiological union as the presence of a bridging callous of two cortices visible on two X-ray views (evidence of the presence of bone healing by A-P plain and lateral view) [22]. Functional results and the complications were evaluated according to the criteria described by Paley and Maar [23]. Activity of daily living scale (ADL) (total score less than 16 was defined as normal function and total score between 16 and 22 was defined as early loss function) and Self-Rating Anxiety Scale (SAS) (total score between 50 and 60 was defined as mild anxiety and total score between 61 and 70 was defined as moderate anxiety while total score more than 70 was defined as severe anxiety) were assessed in follow-up.

### 2.4. Statistical Analysis

All data were processed by SPSS (22.0, USA). Data were presented as median and interquartile range (IQR) for continuous variables and percentages for dichotomous variables. Mann-Whitney *U* test was used for continuous variables. The chi-square test or Fisher exact test was used for dichotomous variables. A multivariate logistic regression analysis was performed to investigate whether internal fixation had adverse effect on the occurrence of complications when compared with external fixation. A *p* value of less than 0.05 was considered significant.

## 3. Results

### 3.1. Clinical Characteristics of Patients in Baseline

A total of 125 patients were included in the present study. The median age was 43.1 years old with 67.2 percentage of male patients. Among them, 41 patients experienced more than 2 operations (including the original surgery for fracture) before the surgery. Patients were divided into internal fixation group and external fixation group according to the standard treatment protocol of senior professor as well as patients' willingness and wishes. The distribution of organisms in the initial irrigation and the improvement of CRP, ESR, WBC, and PCT were shown in Table 1.

There were 57 patients who underwent internal fixation while 68 patients underwent external fixation. There was no difference on age, gender, type of initial fracture, and the methods of skeletal stabilization when admitted to our hospital (Table 2). Patients in external group suffered longer time from injury [11.0 (6.0–15.1) m versus 7.1 (4.0–12.8) m, *p* = 0.012] and had larger bone defect [1.9 (1.3–2.9) cm versus 1.7 (1.0–2.3) cm, *p* = 0.038] compared with patients in internal group. Also, patients in external fixation group had higher body mass index [29.0 (25.9–31.9) kg/m^2^ versus 27.0 (23.5–29.9) kg/m^2^, *p* = 0.004], fasting blood glucose [8.2 (7.0–9.3) mmol/L versus 7.5 (6.4–8.9) mmol/L, *p* = 0.013], and higher level of total cholesterol [4.6 (4.3–4.8) mmol/L versus 4.4 (4.3–4.7) mmol/L, *p* = 0.002] when compared with internal fixation group (Table 2).

### 3.2. Salvage Rate and Related Complications in Follow-Up between Groups

Patients were followed up and assessed at 1.5, 3, 6, 9, and 12 months after operation.

**Figure 1:**
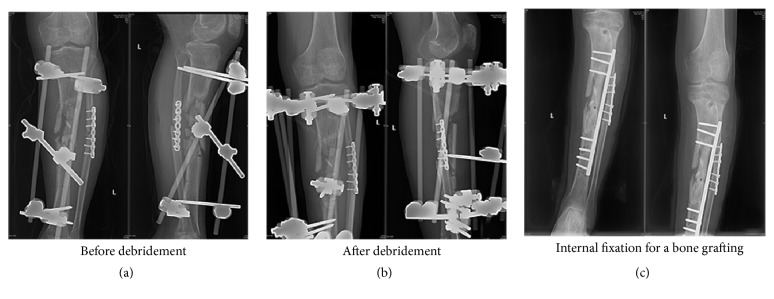
A patient was transferred to our hospital from another institution after external fixation for middle tibia fracture. (a) He presented with an active infection with fistulas and nonunion of tibia shaft. (b) The old external fixer was removed combined with a radical debridement and fixation with another external system. (c) Follow-up X-ray was shown after bone graft with internal fixation.

In internal fixation group, 4 patients experienced infection recurrence (7%) and one of them chose amputation because of economic burden and intolerance of long-time treatment. One patient got refracture (1.8%) without infection after 7 months and we conducted another open reduction and internal fixation, which healed 6 months after second operation. Six patients went on to nonunion (10.5%) in 12-month follow-up. Among them, two underwent amputation and another one died at 2 months because of cardiovascular disorder. Patients had a median union time of 10.6 months and the salvage rate was 94.7%. The ratio of satisfactory functional status was 75.4% (Table 3).  Figure 1 presents the follow-up X-ray images of a patient who underwent bone grafting with internal fixation.

**Figure 2:**
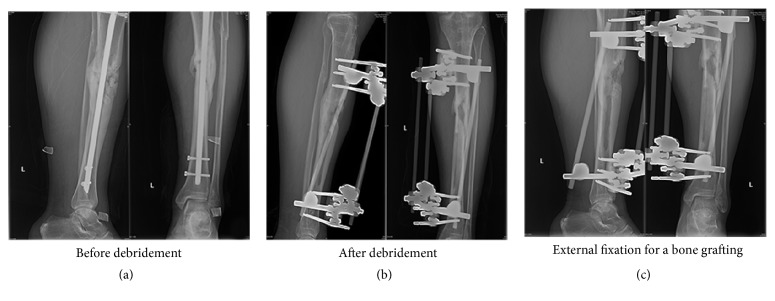
A patient was transferred to our hospital from another institution after intramedullary nailing for upper 1/3 tibia fracture. (a) He presented with an active infection with fistulas and nonunion of tibia shaft. (b) The intramedullary nail was removed combined with a radical debridement and exchanged to external fixation. (c) Follow-up X-ray was showed after bone graft with external fixation.

In external fixation group, 6 patients experienced infection recurrence (8.8%) and all patients underwent a second bone graft with external fixation after sufficient irrigation and debridement procedures. Ten patients went on to nonunion (14.7%) and, among them, three underwent amputation while the rest underwent a second operation. Patients had a median union time of 10.4 months and the salvage rate was 95.6%. The ratio of satisfactory functional status was 78% (Table 3).  Figure 2 presents the follow-up X-ray images of a patient undergoing bone grafting with external fixation.

In ADL assessment, patients in internal fixation groups had 80.7% subjects with normal function while there was only 52.9% subjects in external fixation group. In SAS assessment, the proportions of mild anxiety, moderate anxiety, and severe anxiety were 38.6%, 57.9%, and 3.5% in internal fixation group and 19.0%, 70.6%, and 10.4% in external fixation group. Patients in internal fixation had lower anxiety level compared with external fixation (Table 4).

### 3.3. Logistic Analysis of Risk Factors Related to the Outcome of Reconstruction

In order to investigate whether internal or external fixation following bone grafting had a better outcome of reconstruction, a multivariate logistic analysis was conducted. As shown in Table 5, internal fixation had no significant influence on the occurrence of postoperation complications. Similar results were found in the salvage and satisfactory functional status (Table 5).

## 4. Discussion

In present retrospective cohort study, the efficiency of two reconstruction methods, namely, internal fixation and external fixation, was compared. Patients in external group suffered longer time from injury, larger bone defect, higher body mass index, and higher level of fasting blood glucose and total cholesterol. Internal and external fixation had similar rate of salvage, satisfactory functional status, and complications. There was no significant influence of internal fixation on the occurrence of postoperation complications after multivariate logistic analysis. Meanwhile, patients who underwent internal fixation experienced higher level of daily living scales and lower level of anxiety.

By using wide resection of infected bone, the odds of relapse-free periods in patients with chronic infectious osteomyelitis are improved prominently, but segmental bone defects occurred in aggressive debridement concomitantly. Autogenous bone graft commonly recognized the effective managements in bony defects and fractures with severe comminution and fracture nonunion [24, 25]. The iliac crest is the most common source as its richness in progenitor cells and growth factors as well as limited morbidity for donor site [26]. In present study, autogenous bone graft from iliac crest was used, which was contributed to the relatively high salvage rate and efficiency.

Autogenous bone graft from iliac crest is effective solution for bone defect in tibia but the size of critical bone defect is limited. As recommended by Chinese Association of Orthopaedic Surgeons (CAOS) in 2016 [1], bone defect less than 4 cm was suitable for merely management with autologous cancellous grafting. If the bone defect was larger than 4 cm, specialized reconstruction technique, such as induced membrane (Masquelet) technique, would be suggested. In Masquelet technique, the key step was the application of PMMA and induction of membrane [27]. The involvement of complex construction technique, such as Masquelet technique and Ilizarov technique, made it hard to simply compare the role of fixation because those complex techniques had several key steps which will influence the outcome of construction. In the present study, only patients whose bone defect is less than 4 cm were included, making it easy to compare the role of fixation in the reconstruction of bone defect. We found no significant influence of internal fixation on the occurrence of postoperation complications when compared with external fixation.

Regarded to the way of fixation, external fixation is preferred rather than internal fixation in bone defect fixing or ankle fusing [28]. External fixation had the advantages in getting stable fixation regardless of the position of trauma or infection as well as avoiding dissection through poor soft tissue [29]. Internal fixation methods often required surgical exposure through an invasive technically demanding procedure and had more chance to incur recurrent infection [30]. However, recent studies revealed that there was no difference between external and internal fixation in the reconstruction of bone defect. Moore et al. showed that both external fixation and internal fixation achieved similar rates of limb salvage and final functional status in these patients, as well as similar rates of infection clearance and bony union [31]. Recent meta-analysis compared the postoperative complications between internal fixation and external fixation and found no significant differences in bone healing complications, nonunion, malunion, or delayed union [32]. In present study, no difference was found between external fixation and internal fixation in the ratio of salvage and occurrence of postoperative complications under the circumstance of good control of active infection. Furthermore, patients underwent internal fixation experienced higher level of daily living scales and lower level of anxiety assessed by ADL and SAS questionnaires.

In present study, the baseline characteristics of patients in two groups are similar, making the result comparable and accurate. However, in the present study, only patients whose bone defect less than 4 cm were enrolled, which inevitably made selection bias. However, since the construction techniques were complex and had several key steps for bone defects more than 4 cm, it is very hard to investigate the role of fixation on the outcome with the existed vital influences and limited sample size at present. We will enlarge our sample size of patients whose bone defect more than 4 cm in further study. Also, the follow-up time is relatively short and the data concerning long-time follow-up was difficult to collect; further cohort studies were needed. Furthermore, the surgical protocols may be unfamiliar in western practice. However, in Chinese patients who had bone defects less than 4 cm and controlled infection, both internal and external fixation following autogenous bone graft had good outcomes. This protocol can be considered as alternative form of management for patients with osteomyelitis in western world.

## 5. Conclusion

In conclusion, we explored the efficiency of external and internal fixation in reconstruction of bone defect caused by debridement in Chinese patients and found no difference between two groups under the circumstance of complete control of active infection. Patients who underwent internal fixation experienced higher level of daily living scales and lower level of anxiety. It was relatively safe to use internal fixation for stabilization in osteomyelitis patients whose bone defects were less than 4 cm and infection was well controlled.

## Figures and Tables

**Table 1:** The distribution of organisms and inflammatory markers in baseline.

Parameters		Surgery type		*p* value
		Internal fixation (*n* = 57)	External fixation (*n* = 68)	
Organisms	*S. aureus* (%)	50.8	54.4	0.925
	*S. epidermidis* (%)	26.3	25.0	
	*Enterococcus faecalis* (%)	15.7	16.2	
	Others (%)	7.2	4.4	

Antibiotics duration (week)		5.2 (4.5–6.5)	5.4 (4.5–6.2)	0.589

Before antibody treatment	WBC (×10^9^/L)	13.0 (11.8–14.1)	13.8 (12.3–15.9)	0.003^*∗∗*^
	ESR (mm/1 h)	47.5 (43.5–50.8)	51.4 (48.2–54.5)	<0.001^*∗∗*^
	CRP (mg/L)	21.1 (18.3–23.1)	20.7 (17.5–23.9)	0.921
	PCT (ng/mL)	0.31 (0.25–0.36)	0.32 ± 0.05	0.120

After antibody treatment	WBC (×10^9^/L)	9.7 (8.0–11.2)	9.4 (8.1–10.5)	0.334
	ESR (mm/1 h)	18.2 (16.3–19.7)	17.2 (16.3–19.0)	0.199
	CRP (mg/L)	10.3 (9.4–11.0)	10.0 (8.9–11.1)	0.503
	PCT (ng/mL)	0.09 (0.06–0.10)	0.080 (0.07–0.09)	0.305

Data are presented as median (interquartile range) or *n* (%). WBC, white blood cells; ESR, erythrocyte sedimentation rate; CRP, C-reactive protein; PCT, procalcitonin; ^*∗∗*^*p* < 0.01.

**Table 2:** Clinical characteristics of patients enrolled in baseline.

Parameters	Surgery type		*p* value
	Internal fixation (*n* = 57)	External fixation (*n* = 68)	
Gender (male, %)	63.2	70.6	0.378
Age (year)	42.0 (37.9–48.8)	43.5 (37.0–47.4)	0.862
Body mass index (kg/m^2^)	27.0 (23.5–29.9)	29.0 (25.9–31.9)	0.004^*∗∗*^
Smoker (%)	40.4	54.4	0.117
Diabetes (%)	26.3	30.9	0.574
Fasting blood glucose (mmol/L)	7.5 (6.4–8.9)	8.2 (7.0–9.3)	0.010^*∗*^
Total cholesterol (mmol/L)	4.4 (4.3–4.7)	4.6 (4.3–4.8)	0.002^*∗∗*^
Triglyceride (mmol/L)	1.3 (1.1–1.4)	1.3 (1.1–1.5)	0.110
Low density lipoprotein (mmol/L)	2.8 (2.5–3.1)	2.7 (2.5–2.9)	0.133
Albumin (g/L)	32.5 (31.2–35.6)	33.5 (31.6–35.8)	0.287
Time since injury (month)	7.1 (4.0–12.8)	11.0 (6.0–15.1)	0.012^*∗*^
Bone defect (cm)	1.7 (1.0–2.3)	1.9 (1.3–2.9)	0.038^*∗*^
Anatomic site			0.124
Lower 1/3 (%)	52.6	69.1	
Middle 1/3 (%)	26.3	20.6	
Upper 1/3 (%)	21.1	10.3	
Initial fracture			0.100
Open (%)	77.2	88.2	
Closed (%)	22.8	11.8	
Skeletal stabilization			0.310
Plaster (%)	63.2	54.4	
Internal (%)	14.0	25.0	
External (%)	22.8	20.6	
Donor site			0.068
Bilateral (%)	59.6	75	
Monolateral (%)	40.4	25	

Data are presented as median (interquartile range) or *n* (%).^*∗*^*p* < 0.05; ^*∗∗*^*p* < 0.01.

**Table 3:** Salvage rate and related complications in follow-up interval.

Parameters	Surgery type		*p* value
	Internal fixation (*n* = 57)	External fixation (*n* = 68)	
Surgery time (minute)	152 (137–174)	165 (147–178)	0.149
Salvage (%)	94.7	95.6	0.824
Functional status (%)	75.4	78.0	0.741
Time to union (month)	10.6 (8.1–14.0)	10.4 (8.1–14.1)	0.785
Complications			
Nonunion (%)	10.5	14.7	0.486
Infection recurrence (%)	7.0	8.8	0.711
Refracture (%)	1.8	0	0.456

Data are presented as median (interquartile range) or *n* (%).

**Table 4:** ADL and SAS assessment of patients enrolled.

Parameters	Surgery type		*p* value
	Internal fixation (*n* = 57)	External fixation (*n* = 68)	
ADL assessment			0.001^*∗∗*^
Normal function (%)	80.7	52.9	
Early loss function (%)	19.3	47.1	
SAS assessment			0.031^*∗*^
Mild anxiety (%)	38.6	19.0	
Moderate anxiety (%)	57.9	70.6	
Severe anxiety (%)	3.5	10.4	

ADL: activity of daily living scale; SAS: Self-Rating Anxiety Scale. ^*∗*^*p* < 0.05; ^*∗∗*^*p* < 0.01.

**Table 5:** The multivariate logistic analysis of risk factors related to the outcome of reconstruction.

	Complications		Salvage		Function status	
	OR (95% CI)	*p* value	OR (95% CI)	*p* value	OR (95% CI)	*p* value
Body mass index (kg/m^2^)	1.035 (0.921–1.163)	0.568	1.019 (0.812–1.278)	0.871	0.965 (0.862–1.082)	0.544
Fasting blood glucose (mmol/L)	0.834 (0.587–1.204)	0.333	1.947 (0.832–4.551)	0.124	1.139 (0.800–1.620)	0.470
Total cholesterol (mmol/L)	1.528 (0.187–12.456)	0.692	1.049 (0.017–66.144)	0.982	1.009 (0.131–7.754)	0.993
Time since injury (month)	1.020 (0.928–1.120)	0.684	0.984 (0.810–1.196)	0.871	1.005 (0.917–1.102)	0.908
Bone defect (cm)	0.777 (0.451–1.337)	0.362	0.632 (0.214–1.866)	0.406	1.236 (0.725–2.108)	0.436
Fixation methods						
Internal fixation	1.312 (0.474–3.631)	0.602	1.014 (0.129–7.964)	0.989	1.066 (0.401–2.831)	0.898
External fixation	–		–		–	

## Supplementary Materials

Supplementary Materials Figure S1. The flow chart of selection of patients.
