# Supplementary material for: Comparison of Monolateral External Fixation and Internal Fixation for Skeletal Stabilisation in the Management of Small Tibial Bone Defects following Successful Treatment of Chronic Osteomyelitis
Source: Biomed Res Int. 2017 Nov 26;2017:6250635. doi: 10.1155/2017/6250635 (PMC5733221; doi:10.1155/2017/6250635)
Supplement: Supplementary Materials — Figure S1. The flow chart of selection of patients. [file 6250635.f1.pptx]

## Slide 1
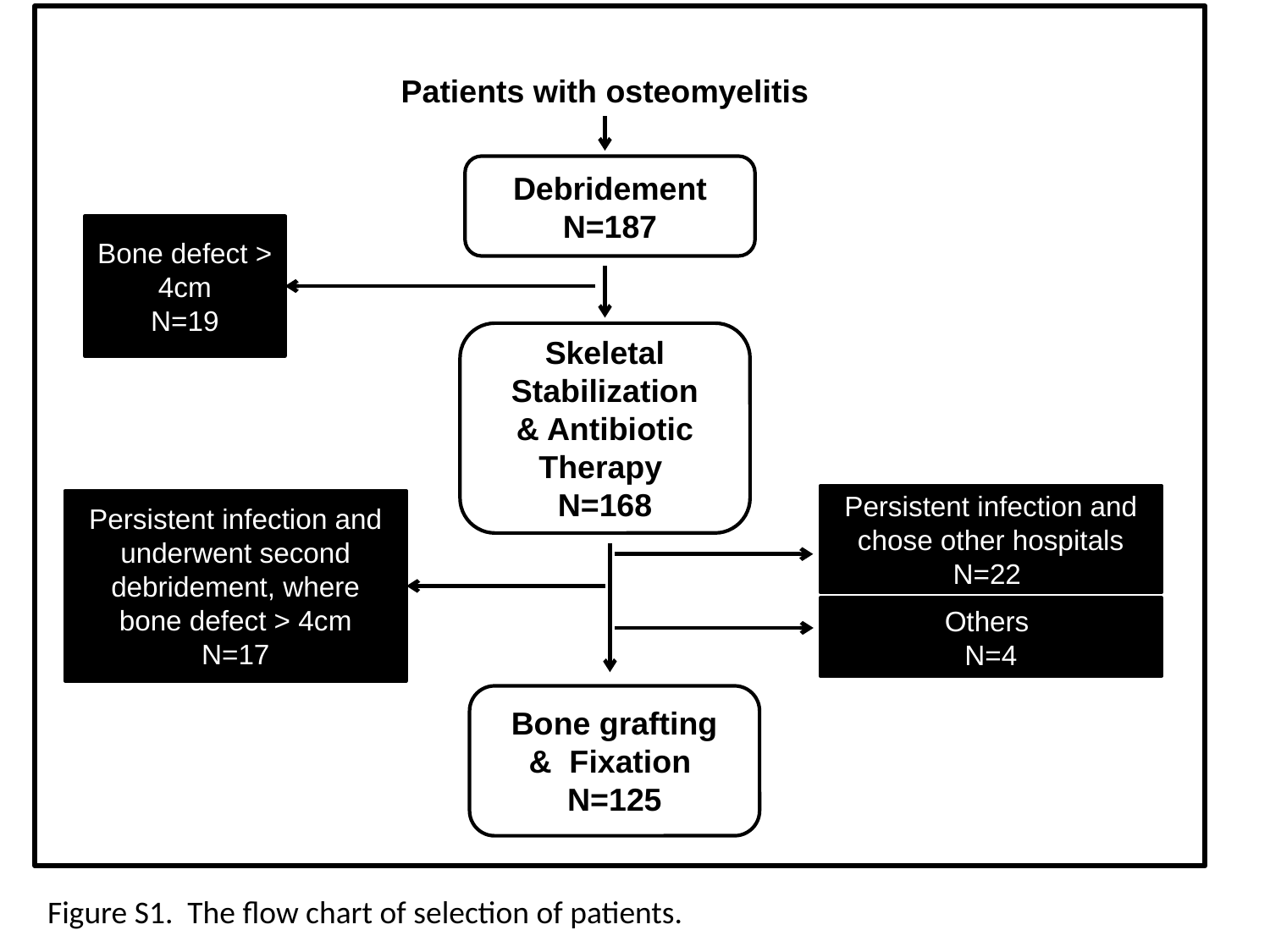

Patients with osteomyelitis
Debridement
N=187
Bone defect > 4cm
N=19
Skeletal Stabilization
& Antibiotic Therapy
N=168
Persistent infection and chose other hospitals
N=22
Persistent infection and underwent second debridement, where bone defect > 4cm
N=17
Others
N=4
Bone grafting
& Fixation
N=125
Figure S1. The flow chart of selection of patients.
